# Research on data transaction compliance: A collaborative and co-governance approach considering buyer erroneous feedback

**DOI:** 10.1371/journal.pone.0335037

**Published:** 2025-10-27

**Authors:** Fanghao Xiao, Xinqing Sun, Junxin Shen, Wenxia Yi

**Affiliations:** 1 School of Marxist, Xiamen Institute of Technology, Xiamen, Fujian, China; 2 School of Management and Economics, Kunming University of Science and Technology, Kunming, Yunnan, China; 3 School of Economics and Management, Southwest Forestry University, Kunming, Yunnan, China; Beihang University, CHINA

## Abstract

Data transactions are frequently hindered by compliance risks due to participants’ lack of self-regulation and the presence weak regulatory mechanism. To address seller’s non-compliant transaction issues, this study proposes a collaborative governance model that integrates platform audits, government oversight, and buyer supervision. This model considers the heterogeneity of buyer utility and applies evolutionary game theory in a noisy feedback environment. The results indicate that accurate buyer feedback can promote compliance and reduce the supervisory burdens on platforms and governments. The reputation effect can enhance the positive behavior of sellers and platforms but has an “inverted U-shaped” relationship with government regulatory enthusiasm. The government’s subsidy and accountability should avoid a “heavy subsidy and light accountability “and the platform’s reward and punishment mechanism should steer clear of “heavy reward and heavy punishment”. Reducing the benefits of government coordination can also curb “free-riding” behaviors.

## 1. Introduction

In the era of the digital economy, data has emerged as one of the most valuable resources of the 21st century. Serving as a crucial link between data providers and consumers, data transactions not only facilitate the effective flow of information but also generate significant economic value for both enterprises and individuals.

As data has become a key factor of production, its role in the economy and society has grown progressively more prominent; consequently, data compliance has emerged as a global concern. Ensuring the compliance of data transactions and establishing an effective collaborative governance mechanism has become a focal point for governments, enterprises, and various sectors of society. However, the rise in data trading activities presents numerous challenges, including privacy protection, data security, and intellectual property infringement.

As the primary responsible party for the legality and compliance of data, the behavior of data providers directly impacts the compliance and security of subsequent transactions. However, some providers with poor self-discipline often exhibit profit-driven tendencies, which are more likely to trigger data security issues. This highlights the current challenges in the regulatory governance system for compliant data transactions and the lack of self-discipline among transaction entities. As a result, there is a need for an efficient governance mechanism that not only addresses but also optimizes and improves governance of compliant data transactions.

## 2. Literature review

In practice, there are two distinct perspectives on the definition of data transaction compliance. In a narrow sense, data transaction compliance refers to the governance system established by the transaction entity to mitigate various compliance risks during data transactions [1]. Broadly speaking, data transaction compliance encompasses a comprehensive governance system for data transactions, aimed at fulfilling the compliance responsibilities of data transaction entities. This system involves third-party data service providers as intermediaries and relies on the enforcement of penalties and incentives by regulatory authorities as guiding principles [2]. The data transaction compliance discussed in this article pertains to a broad interpretation of data transaction compliance.

As data becomes a critical factor of production, scholars have approached the topic from diverse perspectives, leveraging characteristics such as data virtuality [3], exclusivity [4], and non-competitiveness [5], with the goal of enhancing data security and compliance. Advanced algorithms like deep learning [6] and federated learning [7] have been employed to improve safety during data circulation, thereby enriching research in the field of data compliance. However, studies on the institutional framework construction for data compliance remain limited, leaving significant gaps. However, studies related to the development of institutional mechanisms for data compliance have been limited, leading to a noticeable gap in the literature. Therefore, this study aims to contribute to the institutional development of regulatory systems that ensure compliant data transactions, thereby promoting high-quality growth in this domain.

To address frequent non-compliant trading behaviors by sellers in data markets, governments and platforms have emerged as the primary regulators. Their relationship is complex and multidimensional, encompassing both a regulator-regulated dynamic and elements of collaboration. Governments must incentivize platforms to leverage their technological capabilities in developing data transaction infrastructure. Though, some platforms overlook data compliance controls due to cost or technical constraints, while governments may also exhibit limited regulatory initiatives because of budgetary restraints. These issues highlight the deficiencies of current governance mechanisms for compliant data transactions, implying that exclusive reliance on governments and platforms is inadequate to address governance challenges. Additional stakeholders must be engaged to provide robust support and auxiliary functions [8].

As direct users of data, buyers represent a vital societal force capable of contributing valuable external perspectives to compliance oversight through feedback and reporting mechanisms. However, assessments of data compliance often exhibit subjectivity and heterogeneity [9], as they primarily dependent on consumers’ specific perceptions of utility. This implies that buyers’ assessment of compliance may be compromised by subjective biases, due to personal preferences or contextual usage differences. Moreover, the quality and utility of data cannot be observed and perceived prior the transaction, and the information asymmetry between the two parties of transaction may lead buyers to provide biased feedback. To align research with real-world scenarios, governance mechanism must account for such subjectivity-induced biases or deviations in buyer feedback.

Compliant data transactions involve multiple stakeholders, including data sellers, governments, platforms, and buyers. Evolutionary game theory serves as a powerful method for analyzing logical relationships and interest linkages among stakeholders, offering high applicability for exploring value demands and cooperative mechanisms [10]. Furthermore, it enables quantitative analysis, with computer simulations visually illustrating variable relationships and their evolutionary trajectories over time.

Previous studies provide foundational insights. Sun [11] constructed a tripartite evolutionary game model to address replication, dissemination, and resale issues in data transactions, proposing methods to enhance market efficiency and robustness, thereby informing platform governance. Benko [8] developed a tripartite evolutionary game model involving platforms, third-party testing agencies, and government regulators to refine data security evaluation mechanisms, collaborative technical governance, and optimized institutional architectures. Fu [12] established a tripartite game model to investigate dynamic interactions among governments, data developers, and consumers, employing MATLAB simulations to explore the impact of key parameters on system evolution and to improve data risk management and security. While research in data security governance has provided valuable insights for our modeling analysis, existing models predominantly focus on binary decision-making scenarios; often overlook multi-stakeholder participation, leaving a significant gap in literature specifically addressing compliant data transaction governance. With the rapid advancement of data transactions, compliance-driven governance has become a critical concern and is emerging as a pivotal area for future research in this field.

In summary, this paper aims to enhance the design of the compliance governance mechanism for data transactions while supporting the development of the data element market. It focuses on the issue of non-compliant transactions by sellers within the platform transaction model. The study proposes a multi-collaborative co-governance model that integrates platform-specific reviews, government administrative oversight, and buyer-assisted supervision. Additionally, it employs evolutionary game theory and numerical simulation analysis to elucidate the policies and directions for governing data compliance transactions. The key innovation of this paper lies in incorporating potential feedback errors in buyer supervision into the model construction. Through a series of simulation analyses, the data compliance governance from the buyer’s perspective is effectively addressed, significantly enhancing the efficiency of compliant data transaction governance. This provides both practical significance and theoretical value to the research.

## 3. Model building

### 3.1. Analysis of the value appeal and synergy mechanism among multiple subjects

Information asymmetry poses a significant barrier to secure data transactions, and the quality of data sellers varies, which inevitably leads to non-compliant transactions [13]. Consequently, data sellers may adopt one of two strategies: compliant transactions or non-compliant transactions. Trading platforms, government agencies, and data buyers, as governance entities, fulfill their respective roles by employing various mechanisms and measures to regulate non-compliant transactions. Their interests are closely interconnected and mutually influential. The following analysis will explore the value proposition and synergy mechanisms among multiple governance entities in data transaction activities, aiming to support the strategic formulation of governance subjects within the context of evolutionary game theory.

(1) Data Buyers. As consumers of data, buyers expect the information they purchase to be compliant and reliable. However, in actual transactions, due to the inherent uncertainty of data, buyers cannot completely avoid speculative behavior by sellers. In this context, buyers can supervise and protect their rights. If a buyer suspects non-compliance in the data, they may report the issue to the trading platform for feedback. The platform will provide appropriate compensation to the buyer after timely verification. The platform will then provide corresponding compensation after timely verification. If both the platform and the government ignore the buyer’s feedback or delay processing it, the buyer can further expose the not only seller’s non-compliant behavior but also the regulatory inaction of platform’s and government authorities to the public. In this way, all three parties are motivated to avoid non-compliance behavior due to reputation pressure. Therefore, buyers adopt two strategies for data compliance supervision: whistleblowing and feedback, as well as exposure and disclosure.
(2) Data Trading Platforms. As a data transaction intermediary, the platform must create a secure trading environment for both parties involved in the transaction and has an obligation to oversee the transaction activities of data sellers. A rigorously vetted platform can prioritize the exclusion of non-compliant sellers, thereby establishing a compliance security advantage that sets it apart from other platforms and attracts more users to engage in trading, ultimately enhancing its reputation and transaction returns. However, there are variations in the development of platforms, some may reduce their investment in compliance reviews to lower operating costs due to limited economic and technological resources. Consequently, the platform has adopted two strategies regarding data compliance reviews: strict review and relaxed review.
(3) Government Regulatory Authorities. As the administrative regulator of the data element market, the government not only has the responsibility to enhance the overall level of data compliance within society but also bears the obligation to improve social welfare, credibility, and economic benefits. Furthermore, the government has the authority to hold accountable sellers who fail to comply with regulations and platforms that do not conduct thorough scrutiny. It is essential for the government to encourage these platforms to rigorously review data and crack down on non-compliant transactions to ensure the efficient and secure operation of the data element market. However, effective data compliance supervision necessitates significant financial investment in developing regulatory standards and training professional executives. Local governments may face financial pressures, policy constraints, and other factors that can hinder their ability to supervise compliance transactions effectively. Consequently, the government has adopted two strategies for data compliance supervision: active supervision and passive supervision.
(4) Data Sellers. Providing compliant data products constitutes not only a prerequisite for sellers to engage in transactional activities, but also a mandatory requirement under legal and market regulations. However, prior to transactions, data products exhibit dual-sided uncertainty in valuation, accompanied by potential information asymmetry between buyers and sellers. Based on the assumption of rational economic actors seeking to maximize their interests, sellers may be incentivized to adopt non-compliant practices for profit-driven motives, including data fabrication, forgery and tampering, as well as submission of fraudulent quality reports. Consequently, data sellers face two strategic options regarding data compliance in transactions: compliant transactions and non-compliant transactions.

### 3.2. Parameter assumptions

Hypothesis 1: The seller, the platform, and the government are three bounded rational agents in a game. The seller’s strategy space consists of {compliant transactions, non-compliant transactions}, where the probability of compliant transaction is represented by *x* (*0 < x < 1*). The platform’s policy space includes {strict censorship, lenient censorship}, with the probability of strict censorship denoted as *y* (*0 < y < 1*). The government’s strategic space comprises {positive regulation, passive regulation}, where the probability of positive regulation is indicated by *z* (*0 < z < 1*).

Hypothesis 2: Prior to the transaction, the seller will clean, verify, desensitize, and integrate the original data to ensure it becomes a compliant product, incurring a cost (*C*_*h*_) to obtain transaction revenues (*U*_*s*_). Profit-driven sellers may engage in speculative practices during this process to maximize their transaction revenues (*U*_*s*_) at a lower cost (*C*_*l*_). The platform enforces strict censorship and implements a reward and punishment mechanism for sellers’ behaviors. Specifically, sellers will receive rewards (*M*_*s*_) from the platform for compliant transactions, while non-compliant transactions will result in penalties (*F*_*s*_) imposed by the platform.

Hypothesis 3: The platform primarily generates profits (*U*_*p*_) by charging service fees and commissions to both parties involved in the transaction. The platform must conduct modeling analysis, visualization, and other operational steps during strict censorship to filter out sellers engaged in non-compliant transactions and prevent illegal activities, which incurs a higher cost (*C*_*g*_). In contrast, lenient censorship incurs a lower cost (*C*_*b*_), and the process includes a review phase that occurs after receiving feedback from buyers in the later stages, which incurs a review cost (*C*_*e*_). The government actively regulates the implementation of a subsidy and accountability mechanism for platform behavior, meaning that platforms will receive government subsidies (*G*_*p*_), for strictly censoring data-compliant transactions. Conversely, if the platform is lax and allows non-compliant transactions by sellers, both the platform and the seller will be held accountable, with the government imposing a penalty (*R*_*p*_).

Hypothesis 4: Data transactions will bring economic benefits (*U*_*g*_) such as taxes to the government. When the government positively regulation, it needs to set up a special regulatory body and personnel, which incurs a higher cost (*C*_*i*_), and passive regulation pays less cost (*C*_*j*_). The positive regulation of the government and the strict censorship of the platform have formed a collaborative governance, which is conducive to improving data compliant transaction, establishing the credibility of the government, and building the excellent reputation of the platform. The Cobb-Douglas production function [14] form is used to reflect the synergistic relationship between the platform and the government, and the benefits of the two can be expressed as $I_{p}=\partial \gamma V_{p}^{\alpha }V_{g}^{\beta }$, $I_{g}=(1-\partial gammaV_{p}^{\alpha }V_{g}^{\beta }$, α∈[0,1], β∈[0,1]. Among them, the ∂ is the benefit distribution coefficient, and the γ is the synergy coefficient, *V*_*p*_, *V*_*g*_ are respectively the benefits generated by the collaborative governance of the platform and the government.

Hypothesis 5: The buyer assists in supervision with a certain probability (*k*), taking into account the potential for erroneous in the buyer’s feedback. We assume that the probability of accurate feedback from the buyer is denoted as *s*, while the probability of noisy feedback is represented by 1-*s*. Buyers who have concerns about data compliance will report their findings to the platform. The platform’s stringent review process can eliminate transactions that violate quality standards; therefore, only the feedback reported during the platform’s lenient censorship phase needs to be considered. Assuming both the probability and success rate of the review process are equal to 1, the platform will penalize the seller (*F*_*s*_) and require the seller to compensate the buyer (*W*). In cases where the government adopts a passive regulatory approach, the platform’s lenient censorship will result in delays in the review process, with both the review probability and success rate assumed to be 0. If the buyer’s efforts to protect their rights fail, this can further expose the seller’s non-compliant transactions and the regulatory inaction of both the platform and the government, leading to reputational losses for the seller (*L*_*s*_), the platform (*L*_*p*_), and the government (*L*_*g*_). Ultimately, this situation results in compensation (W) being paid by the seller. Table 1 provides standardized definitions for all critical parameters referenced in this study.

**Table 1:** Definitions for all critical parameters.

Parameters	Definitions
*x*	The probability of compliant transaction
*y*	The probability of strict censorship
*z*	The probability of positive regulation
*k*	The probability of buyer supervision
*s*	The probability of accurate feedback from the buyer
*C*_*h*_	Cost of compliant transactions for seller
*C*_*l*_	Cost of non-compliant transactions for seller
*C*_*g*_	Cost of strict censorship by platform
*C*_*b*_	Cost of lenient censorship by platform
*C*_*i*_	Cost of positive regulation by government
*C*_*j*_	Cost of passive regulation by government
*C*_*e*_	Cost of platform verification
*U*_*s*_	Revenues of seller
*U*_*p*_	Profits of platform
*U*_*g*_	Economic benefits of government
*M*_*s*_	Platform rewards for seller
*F*_*s*_	Platform penalties for seller
*G*_*p*_	Government subsidies for platform
*R*_*p*_	Government penalties for platform and seller
*I*_*p*_	The benefits of collaborative governance of the platform
*I*_*g*_	The benefits of collaborative governance of the government
*W*	Compensation received by buyer
*L*_*s*_	Reputational loss of seller
*L*_*p*_	Reputational loss of platform
*L*_*g*_	Reputational loss of government

According to the scenario description and assumptions outlined above, a multi-faceted collaborative co-governance evolutionary game model has been developed, and the benefit matrix is presented in Table 2.

**Table 2:** Matrix of evolutionary returns.

The main body of the game		Government	
		Positive regulation *z*	Passive regulation *1-z*
Seller compliant transaction *x*	Platform strict censorship *y*	*U*_*s*_ * + M*_*s*_ *-C*_*h*_	*U*_*s*_ * + M*_*s*_ *-C*_*h*_
		*I*_*p*_ * + G*_*p*_ *+U*_*p*_ *-M*_*s*_ *-C*_*g*_	*U*_*p*_ *-M*_*s*_ *-C*_*g*_
		*I*_*q*_ * + U*_*g*_ *-C*_*i*_ *-G*_*p*_	*U*_*g*_ *-C*_*j*_
	Platform lenient censorship *1-y*	*U*_*s*_ *-C*_*h*_	*U*_*s*_ *-C*_*h*_
		*U*_*p*_-*C*_*b*_-*kC*_*e*_	*U*_*p*_-*C*_*b*_
		*U*_*g*_ *-C*_*i*_	*U*_*g*_ *-C*_*j*_
Seller non-compliant transaction *1-x*	Platform strict censorship *y*	*-C*_*l*_-*F*_*s*_	*-C*_*l*_-*F*_*s*_
		*I*_*p*_* + G*_*p*_*+U*_*p*_+*F*_*s*_*-C*_*g*_	*U*_*p*_+*F*_*s*_*-C*_*g*_
		*I*_*q*_ * + U*_*g*_ *-C*_*i*_ *-G*_*p*_	*U*_*g*_ *-C*_*j*_
	Platform lenient censorship *1-y*	*U*_*s*_*-C*_*l*_-*ks* (*W + F*_*s*_)-*R*_*p*_	*U*_*s*_*-C*_*l*_-*ksW*-*ksL*_*s*_
		*U*_*p*_-*C*_*b*_-*kC*_*e*_ + *ksF*_*s*_-*R*_*p*_	*U*_*p*_-*C*_*b*_-*ksL*_*p*_
		*U*_*g*_ + *2R*_*p*_*-C*_*i*_	*U*_*g*_*-C*_*j*_-*ksL*_*g*_

The expected return for the seller when selecting a compliant transaction is denoted as *E*_*a1*_, while the expected return for a non-compliant transaction is represented as *E*_*a2*_. The dynamic equation for the copy is *F(x)*, resulting in:


$$\begin{array}{c} E_{a1}=yz(U_{s}+M_{s}-C_{h})+y(1-z)(U_{s}+M_{s}-C_{h})+(1-y)z(U_{s}-C_{h})+(1-y)(1-z)(U_{s}-C_{h})\\ =U_{s}-C_{h}+yM_{s}\\ E_{a2}=yz(-C_{l}-F_{s})+y(1-z)(-C_{l}-F_{s})+(1-y)z(U_{s}-C_{l}-ksW-ksF_{s}-R_{p})\\ +(1-y)(1-z)(U_{s}-C_{l}-ksW-ksL_{s})\\ =(1-y)U_{s}-C_{l}-yF_{s}-(1-y)ksW-(1-y)z(ksF_{s}+R_{p})-(1-y)(1-z)ksL_{s}\\ \overset{―}{E_{a}}=xE_{a1}+(1-x)E_{a2}\\ F(x)=\frac{dx}{dt}=(E_{a1}-\overset{―}{E_{a}})=x(1-x)(E_{a1}-E_{a2})\\ =x(1-x)[yU_{s}+C_{l}-C_{h}+yM_{s}+yF_{s}+(1-y)ksW+(1-y)z(ksF_{s}+R_{p})+(1-y)(1-z)ksL_{s}] \end{array}$$ (1)

The expected return of the platform with strict censorship is denoted as *E*_*b1*_, while the expected return with lenient censorship is represented as *E*_*b2*_. The dynamic equation for the copy is expressed as *F(y)*, resulting in:


$$\begin{array}{c} \begin{array}{c} E_{b1}=xz(I_{p}+G_{p}+U_{p}-M_{s}-C_{g})+x(1-z)(U_{p}-M_{s}-C_{g})+(1-x)z(I_{p}+G_{p}+U_{p}+F_{s}-C_{g})\\ +(1-x)(1-z)(U_{p}+F_{s}-C_{g})\\ =zI_{p}+zG_{p}+U_{p}-xM_{s}-C_{g}+(1-x)F_{s}\\ E_{b2}=xz(U_{p}-C_{b}-kC_{e})+x(1-z)(U_{p}-C_{b})+(1-x)z(U_{p}+ksF_{s}-C_{b}-kC_{e}-R_{p})+(1-x)(1-z)(U_{p}-C_{b}-ksL_{p})\\ =U_{p}-C_{b}-zkC_{e}+(1-x)zksF_{s}-(1-x)zR_{p}-(1-x)(1-z)ksL_{p}\\ \overset{―}{E_{b}}=yE_{b1}+(1-y)E_{b2}\\ F(y)=\frac{dy}{dt}=(E_{b1}-\overset{―}{E_{b}})=y(1-y)(E_{b1}-E_{b2})\\ =y(1-y)[zI_{p}+zG_{p}+C_{b}-xM_{s}-C_{g}+(1-x)F_{s}+zkC_{e}-(1-x)zksF_{s}+(1-x)zR_{p}+(1-x)(1-z)ksL_{p}] \end{array} \end{array}$$ (2)

The expected return of the government when selecting positive regulation is denoted as *E*_*c1*_, while the expected return of passive regulation is represented as *E*_*c2*_. The dynamic equation for the copy is expressed as *F(z)*, resulting in:


$$\begin{array}{c} E_{c1}=xy[I_{g}+U_{g}-C_{i}-G_{p}]+x(1-y)(U_{g}-C_{i})+(1-x)y[I_{g}+U_{g}-C_{i}-G_{p}]+(1-x)(1-y)(U_{g}+2R_{p}-C_{i})\\ =yI_{g}+U_{g}-C_{i}-yG_{p}+(1-x)(1-y)2R_{p}\\ E_{c1}=xy(U_{g}-C_{j})+x(1-y)(U_{g}-C_{j})+(1-x)y(U_{g}-C_{j})+(1-x)(1-y)(U_{g}-C_{j}-ksL_{g})\\ =U_{g}-C_{j}-(1-x)(1-y)ksL_{g}\\ \overset{―}{E_{b}}=yE_{c1}+(1-y)E_{c2}\\ F(z)=\frac{dz}{dt}=(E_{c1}-\overset{―}{E_{c}})=z(1-z)(E_{c1}-E_{c2})\\ =z(1-z)[yI_{g}+C_{j}-C_{i}-yG_{p}+(1-x)(1-y)2R_{p}+(1-x)(1-y)ksL_{g}] \end{array}$$ (3)

## 4. Equilibrium point analysis

The replication dynamic equations of the tripartite evolutionary game are summarized as follows:


$$\left\{ \begin{array}{c} F(x)=x(1-x)[yU_{s}+C_{l}-C_{h}+yM_{s}+yF_{s}+(1-y)ksW+(1-y)z(ksF_{s}+R_{p})+(1-y)(1-z)ksL_{s}nonumber\\ F(y)=y(1-y)[zI_{p}+zG_{p}+C_{b}-xM_{s}-C_{g}+(1-x)F_{s}+zkC_{e}-(1-x)zksF_{s}+(1-x)zR_{p}+(1-x)(1-z)ksL_{p}]\\ F(z)=z(1-z)[yI_{g}+C_{j}-C_{i}-yG_{p}+(1-x)(1-y)2R_{p}+(1-x)(1-y)ksL_{g}] \end{array}\right.$$ (4)

The Jacobian matrix J is obtained by correlation calculation.


$$J=\left(\begin{array}{ccc} @ccc@(1-2x)\left[\begin{array}{c} yU_{s}+C_{l}-C_{h}\\ +(1-y)ksW+yF_{s}\\ +(1-y)z(ksF_{s}+R_{p})\\ +(1-y)(1-z)ksL_{s}\\ +yM_{s} \end{array}\right] & x(1-x)\left[\begin{array}{c} U_{s}+M_{s}+F_{s}\\ -(1-z)ksL_{s}\\ -ksW-z(ksF_{s}+R_{p}) \end{array}\right] & x(1-x)\left[\begin{array}{c} \left(1-y)(ksF_{s}+R_{p}\right)\\ -(1-y)ksL_{s} \end{array}\right]\\ y(1-y)\left[\begin{array}{c} -M_{s}-F_{s}+zksF_{s}\\ -zR_{p}-(1-z)ksL_{p} \end{array}\right] & (1-2y)\left[\begin{array}{c} zI_{p}+zG_{p}+C_{b}\\ -xM_{s}-C_{g}\\ +(1-x)F_{s}+zkC_{e}\\ -(1-x)zksF_{s}\\ +(1-x)zR_{p}\\ +(1-x)(1-z)ksL_{p} \end{array}\right] & y(1-y)\left[\begin{array}{c} I_{p}+G_{p}+kC_{e}\\ -(1-x)ksF_{s}\\ +(1-x)R_{p}\\ -(1-x)ksL_{p} \end{array}\right]\\ z(1-z)\left[\begin{array}{c} -(1-y)2R_{p}\\ -(1-y)ksL_{g} \end{array}\right] & z(1-z)\left[\begin{array}{c} I_{g}-(1-x)2R_{p}\\ -(1-x)ksL_{g}-G_{p} \end{array}\right] & (1-2z)\left[\begin{array}{c} yI_{g}+C_{j}-C_{i}-yG_{p}\\ +(1-x)(1-y)2R_{p}\\ +(1-x)(1-y)ksL_{g} \end{array}\right] \end{array}\right)$$ (5)

According to the equilibrium point stability discriminant method, an equilibrium point is considered asymptotically stable when all the eigenvalues of the Jacobian matrix are negative. Conversely, if there is at least one positive eigenvalue in the Jacobian matrix, the equilibrium point is deemed unstable. The eight pure strategy equilibrium points are substituted into the matrix J, and the corresponding eigenvalues and stability conditions for each equilibrium point are presented in Table 3.

**Table 3:** Eigenvalues and stability conditions of each equilibrium point.

Equilibrium point	Eigenvalue			Stable situation
	*λ*_*1*_	*λ*_*2*_	*λ*_*3*_	
A_1_ (0, 0, 0)	*C*_*l*_ *-C*_*h*_ * + ksW + ksL*_*s*_	*C*_*b*_ *-C*_*g*_ * + F*_*s*_ * + ksL*_*p*_	*C*_*j*_ *-C*_*i*_ * + 2R*_*p*_ * + ksL*_*g*_	Scenario 1
A_2_ (0, 1, 0)	*U*_*s*_ * + M*_*s*_ * + F*_*s*_ * + C*_*l*_ *-C*_*h*_	*C*_*g*_ *-C*_*b*_ *-F*_*s*_ *-ksL*_*p*_	*I*_*g*_ *-G*_*p*_ * + C*_*j*_ *-C*_*i*_	/
A_3_ (0, 0, 1)	*C*_*l*_ *-C*_*h*_ * + ksW + ksF*_*s*_ * + R*_*p*_	*I*_*p*_ * + G*_*p*_ * + C*_*b*_ *-C*_*g*_ * + kC*_*e*_ * + Rp + F*_*s*_ *-ksF*_*s*_	*C*_*i*_ *-C*_*j*_ *-2R*_*p*_ *-ksL*_*g*_	Scenario 2
A_4_ (0, 1, 1)	*U*_*s*_ * + M*_*s*_ * + F*_*s*_ * + C*_*l*_ *-C*_*h*_	*C*_*g*_ *-C*_*b*_ * + ksF*_*s*_ *-I*_*p*_ *-G*_*p*_ *-R*_*p*_ *-F*_*s*_ *-kC*_*e*_	*G*_*p*_ * + C*_*i*_ *-C*_*j*_ *-I*_*g*_	/
A_5_ (1, 0, 0)	*C*_*h*_ *-C*_*l*_ *-ksW-ksL*_*s*_	*C*_*b*_ *-C*_*g*_ *-M*_*s*_	*C*_*j*_ *-C*_*i*_	Scenario 3
A_6_ (1, 1, 0)	*C*_*h*_ *-C*_*l*_ *-U*_*s*_ *-M*_*s*_ *-F*_*s*_	*M*_*s*_ * + C*_*g*_ *-C*_*b*_	*I*_*g*_ *-G*_*p*_ * + C*_*j*_ *-C*_*i*_	/
A_7_ (1, 0, 1)	*C*_*h*_ *-C*_*l*_ *-ksW-ksF*_*s*_ *-R*_*p*_	*I*_*p*_ * + G*_*p*_ * + kC*_*e*_ * + C*_*b*_ *-C*_*g*_ *-M*_*s*_	*C*_*i*_ *-C*_*j*_	/
A_8_ (1, 1, 1)	*C*_*h*_ *-C*_*l*_ *-U*_*s*_ *-M*_*s*_ *-F*_*s*_	*M*_*s*_ * + C*_*g*_ *-C*_*b*_ *-I*_*p*_ *-G*_*p*_ *-kC*_*e*_	*C*_*i*_ *-C*_*j*_ * + G*_*p*_ *-I*_*g*_	Scenario 4

According to the parameter assumptions outlined above, it can be observed that the eigenvalues *λ*_*1*_ of equilibrium points A_2_ (0, 1, 0) and A_4_(0, 1, 1), as well as *λ*_*2*_ of the equilibrium point A_6_(1, 1, 0), and *λ*_*3*_ eigenvalues of equilibrium A_7_(1, 0, 1), are all positive, indicating that these points are unstable. Conversely, there are four stable equilibrium points in the system: A_1_ (0, 0, 0), A_3_ (0, 0, 1), A_5_ (1, 0, 0) and A_8_ (1, 1, 1).

Scenario 1: When the conditions *C*_*l*_*-C*_*h*_* + ksW + ksL*_*s*_* < 0, C*_*b*_*-C*_*g*_* + F*_*s*_* + ksL*_*p*_* < 0, C*_*j*_*-C*_*i*_* + 2R*_*p*_* + ksL*_*g*_* < 0*, A_1_(0, 0, 0) represents the point of gradual stability. The stabilization strategy involves the seller engaging in non-compliant transactions, the platform exercising lenient censorship, and the government adopting a passive regulatory approach. In this scenario, there is a significant deficiency in quality supervision within the data element market, which allows non-compliant transactions to occur, jeopardizing the interests of buyers and disrupting market order. This situation urgently necessitates regulation and governance. The three inequalities indicate that the primary factor inhibiting positive behavior among the parties involved is cost. Additionally, the controllable variables in these inequalities primarily include the buyer’s reporting feedback and exposure disclosure variables, highlighting the importance of buyer supervision in ensuring compliance in data transactions.

Scenario 2: When the conditions *C*_*l*_*-C*_*h*_* + ksW + ksF*_*s*_* + R*_*p*_* < 0, I*_*p*_* + G*_*p*_* + C*_*b*_*-C*_*g*_* + kC*_*e*_* + R*_*p*_* + F*_*s*_*-ksF*_*s*_* < 0* and *C*_*i*_*-C*_*j*_*-2R*_*p*_*-ksL*_*g*_* < 0*, A_3_(0, 0, 1) represents the gradual stability point. The stabilization strategy involves the seller engaging in non-compliant transactions, the platform exercising lenient censorship, and the government adopting a passive regulatory approach. In this scenario, although the government actively fulfills its regulatory responsibilities, its limited authority remains insufficient to rectify the sellers’ non-compliant transactions. However, the government can implement a subsidy and accountability mechanism to encourage the platform to rigorously assess data quality and facilitate the transition of A_3_(0, 0, 1) to the ideal stability point.

Scenario 3: When the conditions *C*_*l*_*-C*_*h*_* + ksW + ksF*_*s*_* + R*_*p*_* < 0, I*_*p*_* + G*_*p*_* + C*_*b*_*-C*_*g*_* + kC*_*e*_* + R*_*p*_* + F*_*s*_*-ksF*_*s*_* < 0* and *C*_*i*_*-C*_*j*_*-2R*_*p*_*-ksL*_*g*_* < 0*, A_5_(1, 0, 0) serves as the gradual stability point. The stabilization strategy involves seller compliance in transactions, lenient censorship by the platform, and proactive government regulation. In this scenario, both the governments and platforms exhibit deficiencies in governing data compliance transactions; however, the seller’s behavior is regulated. The stability point A_5_(1, 0, 0) can be achieved solely by satisfying the requirement *C*_*h*_*-C*_*l*_*-ksW-ksL*_*s*_* *< 0**. Additionally, the buyer’s oversight plays a crucial role in compensating for the governance shortcomings of both the government and the platform.

Scenario 4: When *C*_*h*_*-C*_*l*_*-U*_*s*_*-M*_*s*_*-F*_*s*_* < 0, M*_*s*_* + C*_*g*_*-C*_*b*_*-I*_*p*_*-G*_*p*_*-kC*_*e*_* < 0* and *C*_*i*_*-C*_*j*_* + G*_*p*_*-I*_*g*_* < 0*, A_8_(1, 1, 1) serves as the point of gradual stability. The stability strategy involves the seller engaging in compliant transactions, the platform enforcing strict censorship, and the government implementing proactive regulation. In this scenario, both the government and the platform fulfill their respective responsibilities, while the seller’s actions are also subject to regulation. The variables in the three inequalities indicate that those related to the government and the platform dominate, highlighting the significant roles of platform oversight and government supervision in the governance of data compliance transactions.

In order to further explore how buyers, platforms, and governments can optimize governance within the multi-dimensional collaborative co-governance model, the stable points A_1_ (0, 0, 0) and A_8_ (1, 1, 1) have been selected for numerical simulation and analysis.

## 5. Numerical simulation

### 5.1. Impact of buyer supervision on the system

There are two strategies a buyer can employ to exercise their right of supervision. The first strategy involves reporting and providing feedback to the platform, after which the buyer receives appropriate compensation once the platform successfully conducts a review. The second strategy entails publicly exposing the seller’s non-compliant transactions, as well as the inaction of both the platform and the government, if the initial report and feedback do not yield results. This approach aims to undermine the reputation and image of all three parties involved. Referring to relevant literature and data trading scenarios, the stable point A_1_ (0, 0, 0) path was simulated with the following parameters: *U*_*s*_* = 5, C*_*h*_* = 3, C*_*l*_* = 1, C*_*g*_* = 6,C*_*b*_* = 2.5, C*_*i*_* = 4, C*_*j*_* = 2.5, C*_*e*_* = 1.5, I*_*p*_* = 2, I*_*g*_* = 6, M*_*s*_* = 1, F*_*s*_* = 1, G*_*p*_* = 0.5, R*_*p*_* = 0.5, L*_*s*_* = *2, L**_*p*_* = 1, L*_*g*_* = 1, k = 0.5, s = 0.5,W = 2*. The initial willingness (*x*_*0*_*, y*_*0*_*, z*_*0*_) was selected as (*0.5, 0.5, 0.5*). The influence of the buyer’s reporting feedback and disclosure exposure-related variables on the stability of the system was simulated and analyzed. The values of {*k, s*} were assigned as follows: {0.1, 0.1}, {0.1, 0.9}, {0.5, 0.5}, {0.9, 0.1}, and {0.9, 0.9}, while keeping all other parameters unchanged.

**Fig 1:**
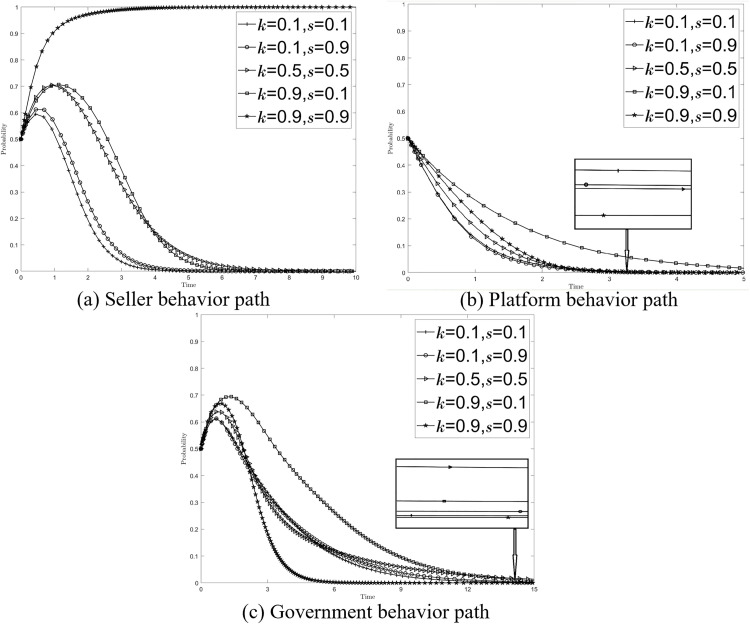
Effect of {*k, s*} on the system.

Fig 1a shows the change of the seller’s behavior path, when the buyer is passive and the erroneous feedback is mostly ({*k, s*}={0.1, 0.1}), the seller tends to the non-compliant trading strategy, if the buyer is encouraged to actively participate in the feedback or take relevant measures to improve the accuracy of the feedback ({*k, s*}={0.1, 0.9},{0.9, 0.1},{0.5, 0.5}), the probability of non-compliance of the seller is reduced, and only the buyer provides positive and accurate feedback ({*k, s*}={0.9, 0.9}), the seller stabilization strategy will change to compliant trading compliance. Fig 1b and 1c show the changes in the behavior paths of platforms and governments, respectively, and the stability strategies of platforms and governments do not change under the different combinations of {*k, s*}, but when buyers provide positive and accurate reporting feedback ({*k, s*}={0.9, 0.9}), the evolution of platforms to relaxed censorship strategies accelerates, and the evolution of governments to passive supervision strategies accelerates. Therefore, it can be speculated that buyer feedback and reporting is an important way to deal with the problem of non-compliant data transactions, and positive and accurate reporting feedback can play the greatest role in governance, promote sellers to comply with the transaction, and at the same time alleviate the cost pressure of platform review and the financial pressure of government supervision.

**Fig 2:**
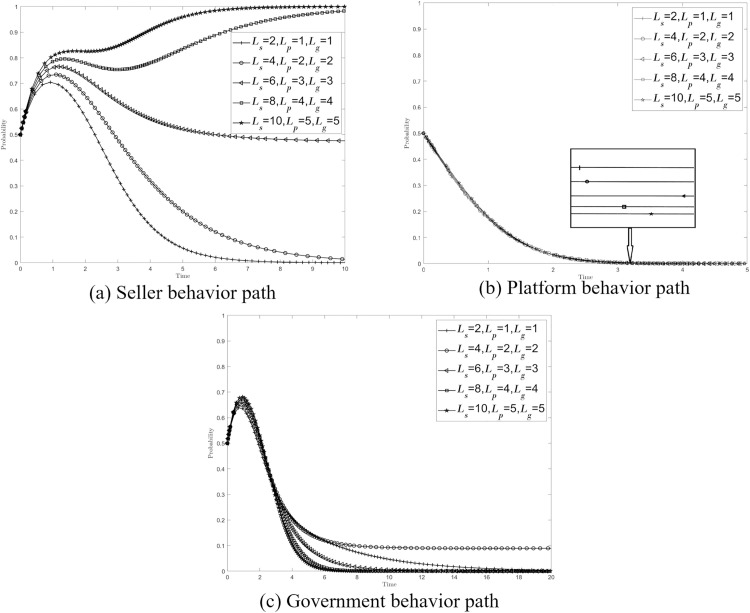
Effect of {*L*_*s*_*, L*_*p*_*, L*_*g*_} on the system.

If the report is fruitless, the buyer can further expose the seller’s non-compliant transactions to the society and the public, expose the regulatory inaction of the platform and the government, and crack down on the bad behavior of the three from a reputational perspective. In order to explore the impact of the reputation loss caused by the buyer’s exposure and disclosure behavior on the stability of the system, the values {*L*_*s*_*, L*_*p*_*, L*_*g*_}={2, 1, 1},{4, 2, 2},{6, 3, 3},{8, 4, 4},{10, 5, 5} are assigned (*L*_*s*_ represents the seller’s reputation loss, *L*_*p*_ represents the platform’s reputation loss, *L*_*g*_ represents the government’s reputation loss), and Fig 2a shows the change of the seller’s behavior path. With the increase of reputational loss, the probability of non-compliant transactions of sellers gradually decreases, and when the reputational loss increases to a certain extent ({*L*_*s*_*, L*_*p*_*, L*_*g*_}={10, 5, 5}), the seller’s stability strategy changes from non-compliant transactions to compliant transactions. Fig 2b and 2c illustrate the changes in the path of platform and government behavior, respectively. With the increase of reputational damage, the stability strategy of the platform and the government has not changed, but the time for the platform to reach the stability strategy of relaxed review has gradually lengthened, and the time for the government to reach the stability strategy of passive supervision has shown a trend of first extending and then shrinking.

Therefore, it can be speculated that increasing the reputation loss can promote the seller’s strategy to change from non-compliant transactions to compliant transactions, which has a certain inhibitory effect on the relaxed review of the platform, but it has an “inverted U-shaped” relationship with the time when the government reaches the passive regulatory stability strategy, that is, it has an “inverted U-shaped” relationship with the government’s regulatory enthusiasm. The reason is that the reputation effect has a restraining effect on the bad behavior of the subject, the seller will take into account word-of-mouth evaluation and other compliance behaviors to improve compliance transactions, and the platform and the government will also take into account the corporate image and social credibility to improve supervision.

### 5.2. The impact of government regulation on the system

**Fig 3:**
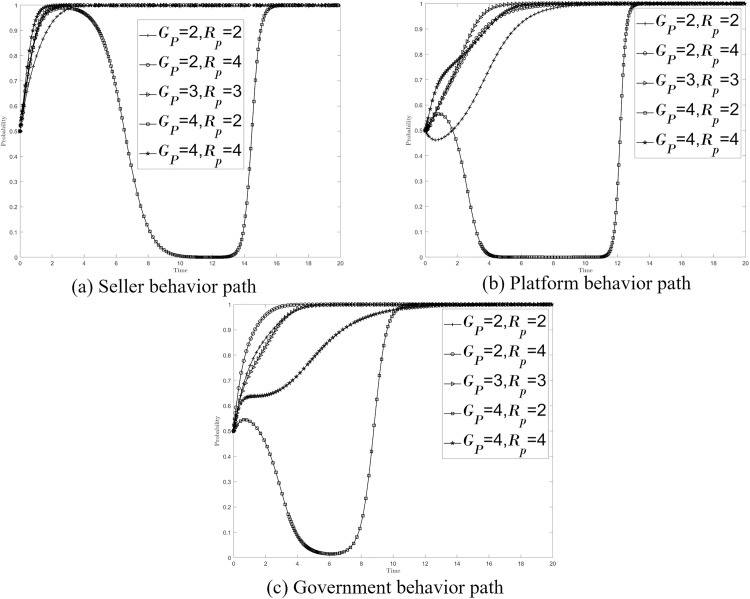
Effect of {*G*_*p*_*, R*_*p*_} on the system.

Government supervision is primarily reflected in the regulation and control of platform strategies, using a subsidy and accountability mechanism to encourage platforms to conduct stringent reviews of data compliance transactions. This prevents speculative sellers from entering the market with non-compliant transactions and, secondly, directly holds non-compliant sellers accountable and takes action against them, thereby enhancing the overall level of data compliance in the trading market. Referring to the relevant literature and the actual situation, the stable point A_8_(1, 1, 1) path was simulated with the following parameters: *Us = 5, Ch = 3, Cl = 1, Cg = 6, Cb = 2.5, Ci = 4, Cj = 2.5,Ce = 1.5, Ip = 2, Ig = 6, Ms = 0.9, Fs = 0.9, Gp = 3, Rp = 3, Ls = 5, Lp = 4, Lg = 2, k = 0.5, s = 0.2, W = 1.5*. Assign values to {*G*_*p*_*, R*_*p*_}={2, 2},{2, 4},{3, 3},{4, 2}{4, 4} in five cases. Fig 3a–3c respectively show the impact of {*G*_*p*_*, R*_*p*_} changes on the behavior path of sellers, platforms and governments, and the stability strategies of the three have not changed.

Notably, the evolution path for {*G*_*p*_*, R*_*p*_}={4, 2} is unique, as it may lead the strategic combination of sellers, platforms, and governments toward or into a state characterized by {non-compliant transactions, relaxed review, and negative supervision}. In this state, the lack of effective market supervision can condone non-compliance, adversely affecting the standardization and security of transactions. Therefore, this state should be avoided as much as possible. Under the model of multiple collaborative co-governance, the government’s subsidy and accountability setting should first avoid “heavy subsidy and light accountability “ (as seen in {*G*_*p*_*, R*_*p*_}={4, 2}). Strategies should then be set according to governance needs: when timely regulation of seller behavior is required, “heavy subsidy and heavy accountability “ can be adopted ({*G*_*p*_*, R*_*p*_}={4, 4}); when platform behavior requires timely regulation, “medium subsidy and heavy accountability “ is appropriate ({*G*_*p*_*, R*_*p*_}={3, 3}) and when there is a need to enhance self-regulatory enthusiasm, “light subsidy and heavy accountability “ can be implemented ({*G*_*p*_*, R*_*p*_}={2, 4}).

### 5.3. Impact of platform review on the system

**Fig 4:**
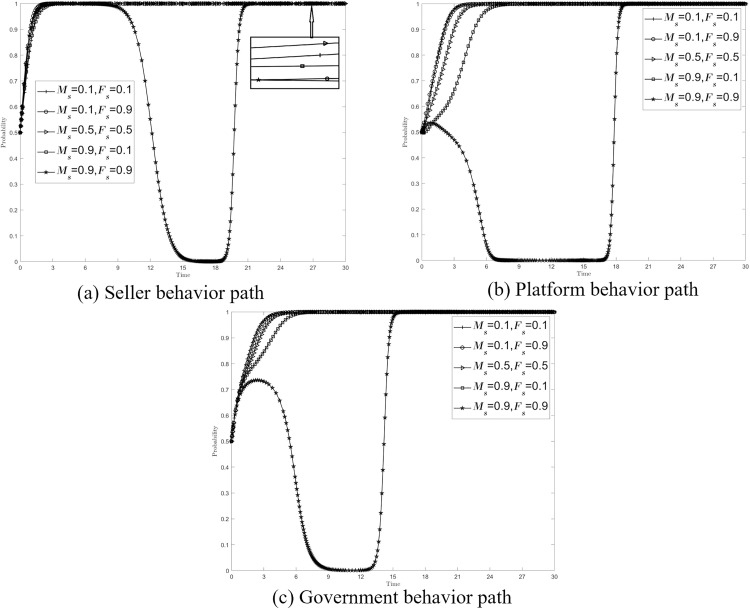
Effect of {*M*_*s*_*, F*_*s*_} on the system.

Platform review is a key procedure to check whether the transaction is compliant or not, and under normal circumstances, the platform will adopt a reward and punishment mechanism to regulate the seller’s transaction behavior. In order to explore how platform review can play the best role in multi-faceted collaborative governance, the initial parameter settings in section5.2 are continued to be used to explore the impact of different combinations of platform rewards and penalties on system stability. Assign values to {*M*_*s*_*, F*_*s*_}={0.1, 0.1},{0.1, 0.9},{0.5, 0.5},{0.9, 0.1}, {0.9, 0.9}. Fig 4a–4c respectively show the impact of {*M*_*s*_*, F*_*s*_} changes on the behavior path of sellers, platforms, and governments, and the stability strategies of the three are unchanged.

Among them, the evolution path of {*M*_*s*_*, F*_*s*_}={0.9, 0.9} is special, and the strategy of sellers, platforms, and governments will be at {non-compliant transactions, relaxed review, and passive supervision} at a certain stage, that is, the gradual stability point evolves to (0, 0, 0), which is the least ideal stability point, and this situation should be avoided. However, the enhancement of rewards and penalties by the platform will not effectively incentivize and restrain the seller’s compliant trading behavior, on the contrary, it will indulge the seller’s non-compliant trading behavior, and affect the platform’s own censorship, and the government will also have “free-riding” behavior. Therefore, under the multi-collaborative co-governance model, the reward and punishment setting of the platform should first avoid “heavy rewards and heavy punishments” ({*M*_*s*_*, F*_*s*_}={0.9, 0.9}), and then set corresponding strategies according to the governance needs: when the seller’s behavior needs to be regulated in a timely manner, “winning the lottery” ({*M*_*s*_*, F*_*s*_}={0.5, 0.5}) can be adopted, and “light rewards and light punishments” ({*M*_*s*_*, F*_*s*_}={0.1, 0.1}) can be adopted to improve their own review attitude and government supervision enthusiasm.

**Fig 5:**
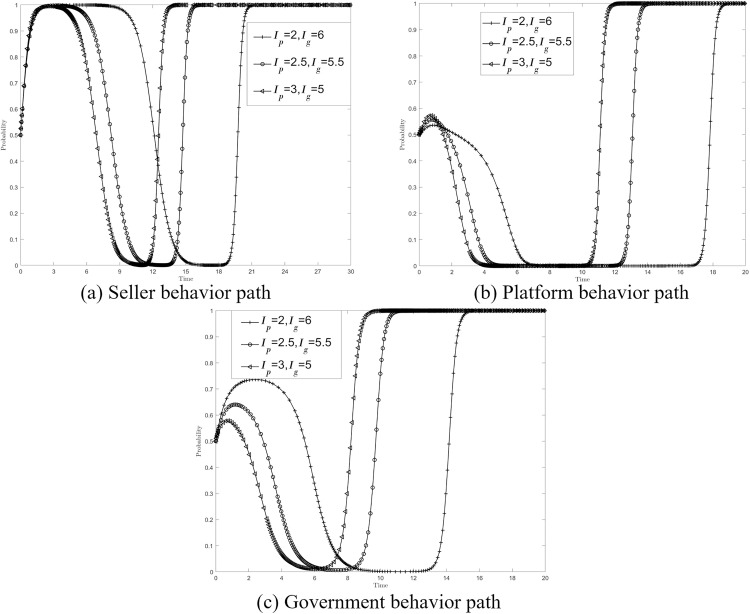
Effect of {*I*_*p*_*, I*_*g*_} on the system.

Based on the above analysis, it can be seen that when the platform strengthens its scrutiny or actively participates in the governance of data compliance transactions, the government is very likely to “free ride”, and the lack of government participation in the market will not be conducive to the whole process of data compliance supervision. On the basis of {*M*_*s*_*, F*_*s*_}={0.9, 0.9}, the values {*I*_*p*_*, I*_*g*_}={2, 6},{2.5, 5.5},{3, 5} are assigned to the three scenarios of {*M*_*s*_*, F*_*s*_}={0.9, 0.9}, respectively, to try to adjust the benefits of collaborative co-governance to prevent the government’s “free-riding” behavior, so as to promote the development of sellers, platforms, and governments towards good behavior strategies. Fig 5a–5c respectively show the impact of the change of collaborative co-governance income on the behavior path of sellers, platforms and governments, and the stability strategies of the three have not changed after increasing the distribution of platform synergistic income, but the time to reach the stability strategy has been shortened. Therefore, it can be inferred that appropriately reducing the government’s collaborative co-governance benefits and increasing the platform’s collaborative co-governance benefits can inhibit the government’s “free-riding” behavior, and at the same time promote the seller’s compliance strategy for compliant transactions and the platform’s strict review strategy.

## 6. Conclusions and suggestions

### 6.1. Conclusions

(1) Buyer supervision plays a crucial role in supporting the governance of data compliance transactions. The use of positive and accurate whistleblowing feedback can enhance supervisory and governance efforts, encouraging sellers to adopt compliant transaction practices. This approach also helps alleviate the cost pressures on platform reviews and the financial burdens on government oversight. The reputational damage resulting from exposure can incentivize sellers to adjust their compliance strategies, thereby exerting a restraining effect on platforms’ lenient review policies. The relationship between this reputational impact and the enthusiasm of government supervision exhibits an “inverted U-shaped” pattern.
(2) Within a model of multi-party collaboration and co-governance, the design of government subsidies and accountability mechanism should first avoid the scenario of “ heavy subsidy and light accountability.” Strategies should then be formulated according to governance priorities: when immediate regulation of seller behavior is required, a “ heavy subsidy and heavy accountability “ approach may be adopted; when it’s necessary to regulate platform behavior promptly, a “light subsidy with heavy accountability “ strategy can enhance the platform’s own regulatory enthusiasm.
(3) The platform’s system of rewards and punishments should initially avoid of “heavy rewards and heavy punishments. Instead, appropriate strategies should be adopted based on governance needs: when timely regulation of seller behavior is required, a “rewards and punish” approach can be implemented; to improve the platform’s review attitude and enhance government supervision enthusiasm, “light rewards and light punishments” may be more suitable.
(4) When the platform strengthens its review processes or actively engages in the governance of data compliance transactions, there is a risk of the government exhibit a “free-rider” phenomenon. If the benefits received by the government through collaborative co-governance are reduced, while those for platforms are increased, it can help eliminate the government’s free-riding behavior. At the same time, such adjustments can promote the seller’s compliance strategies and encourage the platform to adopt stricter review policies.

First, by comparing the compliant data transaction governance model proposed in this study with Sun’s framework for data security governance framework, it is evident that our model introduces a buyer reporting and feedback mechanism. This mechanism effectively compensates for regulatory gaps in governmental and platform oversight, thereby reducing regulatory costs for both entities. Second, in contrast to Fu’s conclusion regarding the unilateral dominance of government subsidies, our findings on accountability-driven subsidy strategies reveal through simulation analysis of subsidy-accountability policy combinations demonstrates that our approach enables the formulation of more precise and targeted policies, this significantly enhancing governance efficacy in compliant data transactions. Finally, this study experimentally adjusts the synergistic governance benefits between governments and platforms to regulate governmental “free-riding” behaviors, yielding promising results in enhancing the standardization collaborative governance mechanism.

To enhance the reliability and applicability of research findings, future studies should prioritize deriving insights from practical implementations. Subsequent research efforts should focus on broadening the investigative scope by actively identifying and collecting real-world transaction cases, particularly those with representative and paradigmatic significance. A systematic analysis of such cases, researchers can extract empirically grounded patterns in the evolution of compliant data transactions and support the development practically viable regulatory governance frameworks.

### 6.2. Suggestions

According to the research results, buyer participation in supervision can effectively fill the governance loopholes of platforms and governments, and help create a good and healthy transaction order. However, in reality, buyers often face problems such as complex reporting procedures and high production costs of feedback materials. Such problems seriously affect the buyer’s willingness to supervise. In this regard, the relevant departments should pay attention to and reflect on it, and take measures to facilitate the buyer’s active participation in the report. For example, intelligent algorithms are used to evaluate the compliance of data products, simplify the steps and procedures of the platform’s review of buyer feedback, and improve the efficiency and accuracy of feedback processing. In addition, the accuracy of the buyer’s feedback content is equally important, while encouraging the buyer to actively participate in the report, it is also necessary to remind the buyer to pay attention to the accuracy of the feedback content, and advocate the buyer to tell facts and have a basis for supervision and reporting through publicity and popular science, so as to avoid the waste of resources and energy of the platform and the buyer caused by invalid feedback.

The data trading platform should take a strict attitude towards data compliance review and create a standardized and orderly data trading venue. In the multi-faceted collaborative co-governance model, the platform needs to formulate reward and punishment strategies according to the seller’s different degrees of non-compliance tendencies. In terms of strategy formulation, the platform can learn from the “1+N” data trading platform group formed by the construction of the Beijing Data Exchange in China, build a service link covering the whole life cycle of data such as data governance, data compliance, data quality evaluation, and data security assessment, improve the data circulation and trading market ecosystem, and also form alliances between platforms of the same type, build data warehouse management, and timely announce sellers or data products with non-compliant transactions to other alliance platforms, so as to clear speculative sellers from the market.

The government should give full play to the responsibilities and obligations of market leaders and actively participate in the supervision and governance of the data trading market. The main methods are: enhancing the seller’s awareness of transaction compliance through media publicity and other means, driving the platform to strictly review data products through subsidy policies, and calling on buyers to actively participate in supervision through legal improvement, so as to improve the overall data compliance level of society. Under the multi-subject supervision model, the government may have a “free ride” phenomenon, which will not be conducive to the compliance supervision of the whole process of data circulation. In this regard, the distribution of collaborative co-governance benefits can be adjusted: reduce the government’s collaborative co-governance benefits and increase the platform’s collaborative co-governance benefits, so as to enhance the enthusiasm of government supervision and prevent the government from “free-riding” phenomenon.

## Supporting information

S1 Fig Effect of {*k, s*} on the system. (TIFF)

S2 Fig Effect of {*L*_*s*_*, L*_*p*_*, L*_*g*_} on the system. (TIFF)

S3 Fig Effect of {*G*_*p*_*, R*_*p*_} on the system. (TIFF)

S4 Fig Effect of {*M*_*s*_*, F*_*s*_} on the system. (TIFF)

S5 Fig Effect of {*I*_*p*_*, I*_*g*_} on the system. (TIFF)
