# Supplementary figures and images for: Research on data transaction compliance: A collaborative and co-governance approach considering buyer erroneous feedback
Source: PLoS One. 2025 Oct 27;20(10):e0335037. doi: 10.1371/journal.pone.0335037 (PMC12558557; doi:10.1371/journal.pone.0335037)

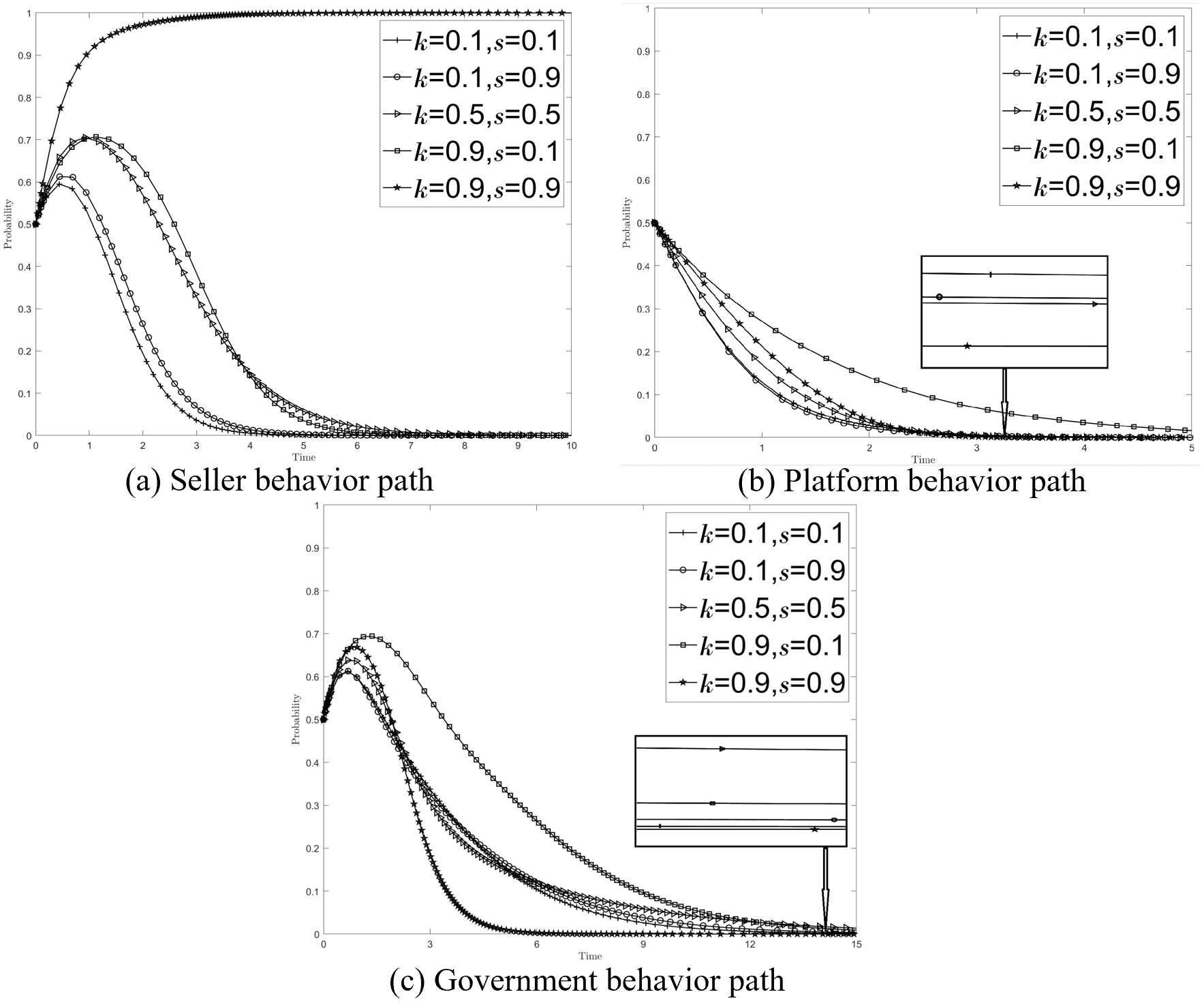

Supplement: S1 Fig — (TIFF) [file pone.0335037.s001.tiff]
